# A shared love: reciprocity and hopefulness in romantic relationships of young adults with chronic pain

**DOI:** 10.3389/fpain.2023.1179516

**Published:** 2023-06-14

**Authors:** Bernie Carter, Abbie Jordan, Paula Forgeron, Pamela Qualter, Holly Saron

**Affiliations:** ^1^ Faculty of Health, Social Care and Medicine, Edge Hill University, Ormskirk, United Kingdom; ^2^ Centre for Pain Research and Department of Psychology, University of Bath, Bath, United Kingdom; ^3^ School of Nursing, University of Ottawa, Ottawa, ON, Canada; ^4^ Manchester Institute of Education, University of Manchester, Manchester, United Kingdom

**Keywords:** chronic pain, relationships, identity, photo-elicitation, young adult, couple (spouses), reciprocity

## Abstract

**Introduction:**

Chronic pain (≥3 months) creates pain-related challenges that may negatively affect how young adults perceive themselves, and, indeed, they often report feeling different compared to peers and prospective romantic partners. Most studies of romantic relationships in young adults living with a long-term condition (including pain), do not consider the perspective of their partner. We present the findings of a qualitative, exploratory interview study (Phase 2 of a mixed methods study). This qualitative phase aimed to explore how young adults with chronic pain and their partners navigate romantic relationships. We focused on how young adults perceive and experience their romantic relationships and the impact, challenges, and benefits associated with living with chronic pain.

**Methods:**

This study used remote (videoconferencing) photo-elicitation interviews with a convenience sample of young adults with chronic pain (aged 18–25 years, UK and Canada) and their partners. Recruitment occurred via social media, pain-related websites and organizations, and professional networks. Five young adults with chronic pain from the UK and Canada formed the e-Advisory Group and provided detailed advice throughout the study. Data analysis used the principles of inductive reflexive thematic analysis to explore the dimensions and meaning of romantic relationships from the views of young adults with chronic pain and their romantic partners.

**Findings:**

Sixteen young adults participated (seven couples plus two young adults with pain who were interviewed without their partner). The young adults with chronic pain were ages 18–24 years (mean 21.88 years, SD 2.23). Four major interpretive themes were generated: Kindred spirits—we just sort of work; Loving in everyday acts—it's not above and beyond, it's concerned supportiveness; It's OK to be vulnerable with each other—we can talk it through; and You can't see over the horizon—hopes and fears for the future.

**Discussion:**

Hopefulness and reciprocity were key to the stories shared by the young adults in the current study. Despite the challenges and limitations imposed by chronic pain, their relationships were characterized by partnership and reciprocity, and they were able to be vulnerable with each other and offer each other support.

## 1. Introduction

As individuals progress from adolescence into young adulthood, social and romantic connections with others (e.g., peers) become more critical (1), with a lack of intimate relationships being associated with reduced levels of well-being across the lifespan (2).

Emerging or young adulthood is the post-adolescent stage of development typically defined as occurring from 18 to 25 years (3, 4). It is a formative time of self-exploration and transition (5) and increasing independence (6). Initiating and sustaining romantic relationships is considered a critical developmental task (3) or milestone for young adults (5). This task can be beset with challenges (7) and rewards (8) with young adults required to integrate their own life plans with those of their romantic partner (9). This milestone can be more difficult to achieve when managing a long-term health condition as young adults with long-term health conditions can experience challenges forming and sustaining romantic relationships due to the constraints of their condition and factors such as poor self-esteem and body confidence (10).

Chronic pain (≥3 months) encompasses both primary chronic pain (a disease entity in its own right), and secondary chronic pain (a key characteristic associated with many long-term physical health conditions) (11). Both types of chronic pain exert similar effects on pain experience and function. Rates of chronic pain increase in adolescence (12), with close to 50% of adolescents with chronic pain continuing to experience pain into adulthood (13). Findings from a systematic review and meta-analysis of young adults (15–34 yrs.) showed an overall prevalence rate of 11.6% for chronic pain among young adults, suggesting that globally 1 in 9 young adults experience chronic pain (14). Other work suggests a range of 5%–30% depending on definitions and other factors (15). Rates of chronic pain continue to increase with age (16, 17).

Chronic pain in young adulthood results in restrictions on daily activities, peer isolation, impacts on relationships with significant others, increased anxiety, isolation from peers and hinders engagement with education and work (15) and has longer term impacts (18, 19). Pain-related challenges (e.g., physical limitations, mood changes due to pain, unpredictable exacerbations) may negatively affect how young adults perceive themselves, and, they often report feeling different compared to peers and prospective romantic partners (20). Young adults with chronic pain report lower-quality romantic relationships (18), whilst evidence demonstrates that living with chronic pain can lead to isolation, helplessness, loss of hope and resentment within adult romantic relationships (21).

Reciprocity has generally been understood as a process of giving and taking (22) and includes an exchange of emotions, thoughts, and behavior. It has long been recognized as a central component of balanced romantic relationships among adolescents (22–24) and among adults from diverse backgrounds (25–28). However, reciprocity may be challenged if, for example, the flow of giving and taking is perceived to be unbalanced as may occur in romantic relationships impacted by chronic pain. The extent to which a person with chronic pain returns the provision of support has been shown to negatively impact relationships (29). In older adults, active engagement (communication about the illness that is done in an open, supportive way encouraging validation of feelings) has been shown to be a positive dyadic coping behavior (30). And, again in older adults, there is good evidence that positive dyadic coping can be indispensable in helping couples to face and adapt to the challenges and demands of a long-term condition together (31).

Studies of romantic relationships of young adults focus almost exclusively on the perspective of the person living with a long-term condition with few engaging with or exploring the perspective of their partner (10). Inclusion of partners is critical in research because romantic relationships are dynamic and reciprocal, and partner dyads can have strong mutual influences on each other's cognitions, emotions, and behaviors (32, 33). Furthermore, many previous studies of young adults have adopted a narrow inclusion criterion, with marriage being seen as a core criterion (34) and do not report the type of relationship the couple are in (e.g., heterosexual or lesbian, gay, bisexual, and transgender (LGBTQ+) (35, 36). Notably, when looking at the literature in this area, it is evident that there is less representation of the voices of young adults with a chronic condition who are in LGBTQ+ relationships (10).

Existing studies have typically adopted a quantitative approach to studying romantic relationships in the context of pain and young adulthood (37), with the aim of identifying general patterns in functioning and behavior. For example, studies have examined the role of focusing on concepts such as relationship quality in young adults living with chronic pain (18). Consequently, the current lack of in-depth detailed qualitative work in this area work means that little is known concerning how young adults perceive romantic relationships in the context of one partner living with pain or what the challenges of engaging in such romantic relationships in this context may be.

Our mixed-methods study aimed to address these knowledge gaps by (i) engaging with young adults with chronic pain in a romantic relationship, defined as “a relationship between two people that extends beyond platonic (just good friends) friendship”, and (ii) working with young adults reporting chronic pain and their partners. We defined chronic pain as “pain that has lasted for 3 months or more, regardless of whether the person has a formal diagnosis”. We used a deliberatively inclusive approach in our work, aiming to encompass young adults in any type of romantic relationship (newly formed through to well-established, state recognized through marriage or civil partnership or not) and heterosexual and LGTBQ+ young adults.

In this paper we present the findings of a qualitative, interview-based study that is aligned with an interpretivist paradigm (38). This qualitative work forms phase 2 of our sequential mixed methods study of young adults with chronic pain and their partners that asked the question “how do young adults with chronic pain and their partners navigate romantic relationships”?

## 2. Methods

We aimed to explore how young adults with chronic pain and their partners navigate romantic relationships, with specific focus on their perceptions and experiences of their relationships and the impact, challenges and benefits associated with living with chronic pain.

### 2.1. Study design

This exploratory, interpretive qualitative design used remote (videoconferencing) photo-elicitation interviews with young adults with chronic pain and their partners, positioning them as experts of their own experience.

The study was approved by the Health-Related Research Ethics Committee (ETH2021-0227), Edge Hill University, UK; the Psychology Research Ethics Committee (21-237) (University of Bath, UK); and the Health Sciences and Science Research Ethics Board (H-10-21-7549), University of Ottawa, Canada.

This study followed the Consolidated Criteria for Reporting Qualitative Research (COREQ) guideline (39).

### 2.2. Public and patient involvement and engagement (e-Advisory group)

The study was guided by an e-Advisory Group of five young adults with chronic pain (aged 18–25 yrs.) from the UK and Canada. They contributed their ideas about the focus of the study and provided crucial input to the study design, particularly in the selection of the measures used in the survey (Phase 1) and in the development of the interview questions. The Advisory Group also provided clear guidance on wording of recruitment flyers, study logo, information sheets and what platforms to use to advertise the study.

The e-Advisory group's contribution to the qualitative phase of our study included supporting the design of the questions, providing us with more sensitive ways of asking a question, suggesting ways in which we could probe more deeply. They continue to provide vital support in the development of dissemination materials.

### 2.3. Participants and setting

Convenience sampling was undertaken from a population of young adults with chronic pain (aged 18–25 years) who had participated in an online survey concerning the nature of their romantic relationships in the first phase of the study. These young adults were from the UK and Canada and had been recruited via social media (a designated Instagram page, and professional Twitter accounts), pain-related websites and organizations, and professional networks. Our sample size was not predetermined but based on the young adults from Phase 1 who agreed to participate in Phase 2 interviews. Our aim was to achieve “information power” (40) rather than consider data saturation.

Young adults with chronic pain who had completed the quantitative online survey in Phase 1 of the study, and who confirmed interest in taking part in an interview, along with their partners (aged ≥18 years) who agreed to be contacted, were sent tailored information sheets by email. The information sheet indicated that either partner could participate even if the other partner was not interested. We had anticipated recruiting 10–20 dyads via our survey, but survey completion was lower (*n* = 68) than the planned for 130 respondents.

Those who indicated interest in taking part in this qualitative study were contacted via email (a copy of the information sheet and consent form were enclosed). Any questions potential participants had were initially answered by email. A mutually convenient time for the video interview was arranged. Prior to beginning the video interview any further questions about the study were answered and then consent was gained prior to starting the interview. Interviews took place between May 2022 and August 2022.

### 2.4. Data collection

Secure voice over internet protocol platforms (Teams or Zoom) were used to conduct the online videocall interviews by the final author (HS). This format facilitated long-distance/remote participation, was economical, convenient, and suitable for sensitive topics (41) and complied with COVID-19 data collection restrictions. We aimed to undertake synchronous interviews with the couple to facilitate their ability to support, question, and supplement each other's perspectives and generate a shared consideration of aspects (e.g., challenges, reciprocal actions) of their relationship.

Photo-elicitation (PE) interviews (42) were used as a trigger for the online, in-depth, conversational interviews. PE was adopted to facilitate a participant-centered interview as it gave participants the opportunity to take photographs or identify images that epitomized the best and difficult/stressful aspects of their romantic relationship. The images could be shared in advance via a secure server. At the start of the interview, the interviewer (HS) invited the participant(s) to talk about 3–4 images they had selected. Although the images were the trigger for the conversation, the interviewer also had a list of questions and prompts they could cover. These were broadly similar to the questions and prompts used in the semi-structured interviews with those participants who chose not to engage with PE. The interview guides aimed to elicit their perceptions, experiences, understanding, benefits, and challenges of navigating their romantic relationship. The guide included questions such as: When you met [name of person] did they have chronic pain? How would you describe your romantic relationship? Does the experience of living with/living with a partner who has chronic pain bring you any benefits as a couple or as individuals? (see Supplementary File for Interview Guide).

At the end of the interview, we sent a Helpful Debrief Sheet that included directions to well-being and pain-related resources that provide help, advice, and support.

### 2.5. Data analysis

All interviews were digitally audio-recorded and transcribed verbatim by a professional transcription service approved by the first author's University. Transcripts were not returned to participants for checking as this is inconsistent within an interpretivist paradigm (38, 43). Interview data were analyzed by the research team (BC, HS, AJ, PF; all female academics; two nurses, one psychologist, one social scientist) in accordance with the principles of inductive reflexive thematic analysis (44). Initial codes, concepts and themes were proposed by BC and shared with the rest of the team prior to detailed discursive conversations about this initial work. The codes and eventual themes were iteratively developed through contributions, reflexive notes, challenges, and new ideas from the team. This allowed coder agreement to be built and refined iteratively based on team consensus. Thus, some initial codes that were proposed by BC were scrapped and replaced by more elegant and robust interpretations. A log (audit trail) was kept of the interpretative shifts in our coding and analytical decision-making. The inclusion of team members in the analysis aligned with an interpretivist paradigm to increase the hermeneutic circle (45) and ensured that the themes were grounded in the data (46). Additionally, we shared preliminary codes and findings with our e-Advisory Group who provided detailed responses to our analysis and presentation of themes; their affirmation of codes and themes and their suggestions for refinement of phrasing of the findings was crucial to our thinking. We incorporated many of their ideas into our final analytical work and this provided us with assurance that our interpretation of the data reflected the perspectives and experiences of the participants.

Pseudonyms were selected for all participants. Each young adult with chronic pain was named alphabetically (A–I). The names of young adult with chronic pain and their partner start with the same letter. The partner of the young adult in chronic pain, as relevant, was indicated by using a superscript “P”, e.g., Billy^P^ is that specific participant's partner.

We addressed the integrity of our qualitative endeavor in two main ways. First, potential biases and assumptions that may have influenced all aspects of the study (design through to analysis and interpretation and beyond) were acknowledged and challenged during iterative oral dialogue and written comments (e.g., all researchers were female and White). The diversity of disciplinary/academic background (as noted earlier) may have been helpful here. Second, individuals wrote reflexive notes, and those contributed to team discussions which in turn helped ensure transparency of the interpretative decision-making process and allowed clarification as needed. Quotations were selected from across the sample to ensure that diversity of experience is presented (47).

## 3. Findings

Sixteen young adults participated; of these, seven couples (young adult with chronic pain and their partner) took part. Two young adults with chronic pain were interviewed without their partner; of these, one partner was reported as unable to join because they were working; the other did not provide a reason for non-participation.

The young adults with chronic pain were ages 18–24 years (mean 21.88 yrs., SD 2.23). Neither the age nor gender of the partner was requested (although to be eligible, partners had to be 18 years or older). None of the couples were married or in a formalized partnership (e.g., civil partnership). Interviews lasted 18–62 min (38 min on average).

Illustrative quotations are used throughout to support the findings. Table 1 provides an overview of the participants in relation to their demographics and key aspects of their relationship. Table 2 provides an overview of the photographic triggers.

**Table 1:** Characteristics of young adults with chronic pain (YAwCP) and partners.

YAwCP	Partner	Location	Pain-related diagnoses	Duration of pain	Age of YAwCP	Gender of YAwCP*	Sexual orientation of YAwCP	Duration of current romantic relationship	Current living circumstances
Ali	Did not participate	Spain, originally from UK	Endometriosis, adenomyosis, arthritis	>5 years	22	Female	Heterosexual	Over 3 years	Living with partner
Brianna	Billy	UK	Joint Hypermobility Syndrome	>5 years	19	Female	Gay Woman/Lesbian	1–3 years	Living in University halls
Christie	Charlie	UK	Endometriosis, hypermobility	1–<2 years	20	Non-binary	Bisexual	Over 3 years	Living with parents
Daisy	Dylan	UK	Back pain (no formal diagnosis)	>5 years	24	Female	Heterosexual	Over 3 years	Living with partner
Ellie	Evan	UK	Lipoedema, lymphoedema, Ehlers Danlos Syndrome, neuropathic pain, Chiari malformation	>5 years	24	Female	Pansexual	7–11 months	Living with parents
Flo	Finlay	Canada	Endometriosis, chronic pelvic pain, nerve pain	>5 years	22	Female	Heterosexual	Over 3 years	Living with partner
Grace	Gabriel	UK	Unprovoked vulvodynia, provoked vestibulodynia	>5 years	24	Female	Bisexual	1–2 years	Living with partner
Hazel	Harry	UK	Juvenile idiopathic arthritis	>5 years	18	Genderfluid	Unlabelled	1–3 years	Living with parents
Indi	Did not participate	UK	Endometriosis	>5 years	24	Female	Heterosexual	Over 3 years	Living with partner

*Female is used to reflect responses in survey to option “Woman/female (including transwoman)”.

**Table 2:** Overview of photographs shared by participants.

Couples	Number shared	Description of photographs shared
Ali	0	
Brianna & Billy^P^	4	Brianna and Billy^P^ a month before they started their relationship Billy^P^ a day before the second COVID-19 lockdown Dock area in a city Sunset over a city
Christie & Charlie^P^	3	A selfie of Christie and Charlie^P^ together smiling Christie standing with a castle in the background Charlie^P^ on one knee proposing to Christie who is sat on a swinging bench seat
Daisy & Dylan^P^	0	
Ellie & Evan^P^	2	An image from the internet of a restaurant with a certain type of chair and clocks on the wall. Ellie and Evan^P^ together at a graduation.
Flo and Finlay^P^	4	Finlay^P^ holding a chocolate cake the couple made together Flo and Finlay^P^ on their bikes Flo sat next to Finlay^P^ with her head on his shoulder and Finlay^P^ has his arm around Flo Finlay^P^ giving Flo a pedicure, painting her toenails
Grace and Gabriel^P^	6	An image of a busy calendar depicting (chosen by Grace). An image of an empty calendar (chosen by Gabriel^P)^ An intimate cocktail bar with whimsical kitchenware and eclectic décor where Gabriel^P^ played gigs Beach with white cliffs behind. An attic room like Gabriel^P^'s University bedroom A house, not discussed in the interview
Hazel & Harry^P^	3	The couple lying in bed, Hazel lying on Harry^P^'s chest and Harry^P^'s arm around Hazel. The word “cute” overlays the image. The couple standing together with red hair-dye on their hair. The word “cute” overlays the image. Hazel on Harry^P^'s back, in the park, Harry^P^ with a big smile on their face. Happy face emojis overlay the image.
Indi	0	

Four major interpretive themes were generated from the analysis of the data.

• Kindred spirits: we just sort of work.
• Loving in everyday acts: it's not above and beyond, it's concerned supportiveness.
• It's OK to be vulnerable with each other: we can talk it through.
• You can't see over the horizon: hopes and fears for the future.

Whilst each of these has a distinct character and focus, they each inform and relate to each other. For a summary of the key components of each theme see Table 3.

**Table 3:** Overview of themes and key components.

Theme	Summary of key components	Condensed exemplar quotes
Kindred spirits: we just sort of work	Connection and closeness	“we were kind of glued together at that point”,
	Reciprocity	“we … seem to have built a closeness that we can both be happy with”
	Learning about partner's pain	“I think the longer we've been going out, it feels a bit more of a shared experience and a shared kind of condition in a way”
	Respecting limitations	“I think it [pain] makes us prioritize what we do want to do, even from just an energy standpoint”.
Loving in everyday acts: it's not above and beyond, it's concerned supportiveness	Living with pain is reciprocal work	“[pain creates] more work, and yet, we're really happy to do [the work]”
	Accommodating pain through everyday acts of loving	“[I’m not] going above or beyond [but] just doing what I feel any partner would or should do”
	Concerned supportiveness	“it was just it was a really sweet action… and it was kind of a dark time…. definitely uplifting”.
	Asking for help and asking to help	“[the] best way to find out what you can do to support your partner with pain is just to ask”
It's OK to be vulnerable with each other: we can talk it through	Being (repetitively) vulnerable	“romance comes [with] a lot of vulnerability and stuff”
	Being open and talking about pain	“[we’re] more confident to share things with each other, …nothing else seems as big as an issue [as pain]”
	Disclosing pain	“how do I go about telling somebody I’ve got all these illnesses and how do I go about saying that the pain I’m in”
You can’t see over the horizon: hopes and fears for the future	Hope for the future as a couple	“[we’re] in a new kind of [exciting] phase of life
	Fears of being a burden	I wouldn’t want anything that you have to deal with to get worse… [although] we [would] be able to deal with as we go”.
	Fears about employment	“making enough money to actually survive while also not wearing myself into the ground”
	Fears about fertility	“if we have to go through IVF we will go through IVF…as a team”

### 3.1. Kindred spirits: we just sort of work

The young adults all talked of the strong sense of connection, closeness, love and being meant for each other. They recalled the time when they had fallen in love:

*I was kind of like, looked to you. And I was like not to get too mushy. But I really loved you (Gabriel^P^)*.

Typically, they described their relationships as “very normal, really happy” (Flo), “just a lovely relationship…a loving one” (Ellie), based on being “comfortable with each other” (Daisy) and sharing “a lot of the same values and a lot of the same interests as well, we're both kind of old souls” (Flo). They often talked about being content that their relationships were characterized by “doing mundane things together… doing little things” (Brianna), enjoying “just existing together” (Billy^P^) rather than needing to engage in very “exciting” and/or spontaneous activities to which pain could be a barrier:

*I have no problem [about not going out spontaneously], as Ellie knows that I don't really like doing things spontaneously anyway (Evan^P^)*.

The closeness they felt was something they had either felt immediately, “we were kind of glued together at that point” (Brianna) or built more gradually over time “we … seem to have built a closeness that we can both be happy with” (Evan^P^). This closeness was reciprocal.

For some couples, this was their first romantic relationship, and this brought with it a sense of excitement and “learning how to be in a relationship” (Flo). Some young adults with chronic pain implied that their pain and/or underlying condition might act as a barrier to establishing a romantic relationship as Ellie explained:

*God bless him [partner] is like the first person I've ever been in a relationship with ….I had no expectations that this would lead to anything (Ellie)*.

The young adults with chronic pain anticipated that their pain might be a burden and a barrier to forming a romantic relationship as they did not “want to let [partner] down” (Ali) or be “a burden and a drip…. [or] having to justify if I’m in pain” (Ellie). In contrast, their partners did not see pain as a barrier to becoming a couple:

*I don't mind being around [Ellie] when she's unwell …., it's like, it's a bridge that we're going to have to cross at some point regardless …. it bothers her more than it bothers me in that sense. But it's just something that I'm sure just through time and experience we'll sort of get more used to (Evan^P^)*.

Learning about and developing an understanding about their partner's pain was noted to be “one of those things that develops over time” (Charlie^P^) but understanding was acknowledged as not being the same as experiencing pain. Despite the literal inability of partners to experience the other's chronic pain, for some, the longevity of a relationship resulted in the experience of pain becoming an increasingly shared experience, mirroring the impact on the couple rather than the individual. However, this understanding helped the young person with chronic pain to see that their pain was not the most important aspect of their relationship:

*I think the longer we've been going out, it feels a bit more of a shared experience and a shared kind of condition in a way (Grace)*.

Respecting any limitations associated with their partner's pain and/or condition was not perceived as being their “fault… you can’t help it. Why get annoyed with you?” (Charlie^P^). This perspective was reassuring, positive and strengthened their relationship:

*I would rather slow down and do what's needed to be with you, rather than go off on my own. Any time with you is always appreciated. So why would I feel bad about that or disappointed? …… I still get to spend time with you (Harry^P^)*.

Pain sometimes took the spontaneity out of life and needed to be worked around and could restrict activities. Charlie^P^ explained that “I think it [pain] makes us prioritize what we do want to do, even from just an energy standpoint…. Let's make sure we do that one thing”. At other times thought had to be put into selecting a “safe space, safe place” (Ellie) to go out for a meal where the seating was comfortable for her, as this could mean they could be there “for quite a long time, spend more time together [there]” (Evan^P^). Pain sometimes required careful scheduling of events and outings to try and reduce the risk of pain being a spoiler. Participants living with pain described this complex scheduling as requiring “a lot of mental kind of gymnastics” (Grace), to facilitate valued time to be spent together in the most convenient manner.

### 3.2. Loving in everyday acts: it's not above and beyond, it's concerned supportiveness

Despite their love for each other and their reluctance to let pain foreshadow their lives, pain played a role in young adults’ relationships and in the lives they led. Often pain was described as a separate entity, as something that is not only independent from the young adult with pain but apparently also independent from the couple.

The young adults often talked in a pragmatic way about “the pain”, pushing it into the background without downplaying its significance to them as a couple. Billy^P^ explained “it's just one of those things, isn't it?” and any compromises were perceived to be things that might take “more work, and yet, we're really happy to do” (Gabriel^P^) and that together they just “have to ride it out, because it doesn’t go away” (Indi).

The young adults, particularly those with chronic pain, talked of their relationships as being “very safe and secure” (Ellie) and characterized by a depth of trust “beyond what I’ve experienced before” (Grace) Although creating a safe and secure relationship might be expected to be something that focused on supporting the young adult with pain, the couples talked of how this reciprocity “worked”, with each of them making the effort to look after their partner. As Hazel explained “if either of us is struggling, then hugs help and just being close” and Finlay^P^ emphasized:

*I didn't have any concerns about taking care of her [Flo]. I mean, I just figured it's what all couples do. Like you look after each other. She's taking care of me right now. So, it's what we do (Finlay^P^)*.

When pain needed to be accommodated due a flare in intensity or due to side effects of the pain or medication such as fatigue or when safety and security was threatened, then partners demonstrated reciprocity in their love for each other through “everyday acts of loving”. These everyday acts were not perceived to be “going above or beyond [but] just doing what I feel any partner would or should do” (Dylan^P^). Grace and Gabriel^P^ talked of these reciprocal acts as a form of “concerned supportiveness”. Grace explained this was underpinned by Gabriel's^P^ sense that “everything is, is fine, as long as I feel fine”.

Everyday acts of loving included a wide range of practical acts such as providing hot water bottles, ice packs, carrying bags, help with hair washing and fetching drinks. Flo and Finlay^P^ described how Finlay^P^ giving Flo a pedicure helped them during a difficult time in the pandemic:

*Finlay^P^: She was going through pain and struggling. So, I just wanted to help her a little bit. And it's just a small thing I could do to help her…*.

*Flo: I think it was just it was a really sweet action… and it was kind of a dark time, so having a little something like that it was definitely uplifting*.

Other acts included ones that demonstrated a non-demanding, non-judgmental physical closeness “he hugged me and it made me feel so much better about what I was feeling and how my day went” (Hazel) and Ellie explained:

*we lay on the bed and I just had my duvet up to me and was like ‘I’m so sorry. I'm in pain. This is part of my [many] conditions. And he just listened to me. And he let me have that cry and he gave me that space without judgement (Ellie)*.

Their partners were aware that despite wanting to be helpful and supportive, and having an insight into what actions they could take, chronic pain could not be fixed. Billy^P^ noted that “sometimes… I wish I could make it better, fix… not fix it, but sometimes it does make me a bit sad…[but] it's not my responsibility to fix it”. Even though pain could not be fixed, actions could help as Harry^P^ explained:

*most of the time it's something that I can't actually solve or don't know how to answer. You always seem even the tiniest bit better just by getting it out [talking] (Harry^P^)*.

There was agreement among the young adults that the “best way to find out what you can do to support your partner with pain is just to ask” (Christie).

Concerned supportiveness was also evident in the way for those couples who could not have sex or where sex took planning, because of the pain that it engendered. Couples worked around the limitations that pain and/or the condition imposed on their sex life. The young adults with chronic pain tended to perceive this as negative and something that created a sense of insecurity or “guilt… like you might not be able to…fulfil all the … aspects of someone's life” (Grace). However, their partners seemed pragmatic about the situation as can be seen in the different perspectives in the interchange between Flo and Finlay^P^:

*Flo: I can't have sex. … I'm like, “Oh, my God, … if it was another girl, she'd be fine, she'd be able to”. But it doesn*'*t seem to bother him too much. …. it's still something kind of difficult to work through.… we*'*ve had really extensive conversations about it. And I know that he's completely patient with me, and I'm open about it…*.

*Finlay^P^: I mean there have been times where I have thought about it, but I don't think I worry about it as much as she does…. I've never had sex. So I don't really know what I'm missing. So it's kind of hard to miss something I've never experienced. So, and I and I enjoy being with her. So, I think in the end, it all kind of makes it positive*.

Sex could not be spontaneous for Grace and Gabriel^P^ and despite Grace's initial concerns about how sex would go the first time they tried, they had a plan which involved scheduling, maximising the support from the medication and a back-up plan with a bottle of wine:

*Grace: I use lidocaine, so then you have to work out, “oh need to take it at least four days before, before we plan to have sex”, then it's really a lot of kind of scheduling…*

*Gabriel^P^: So the first time you were like, “Okay, let's try it, it might work but also, I've bought a bottle of wine just in case” …you don't want to start and then stop…*

*Grace: Yeah, I didn't want like the memory of it to be shit so I thought let's get a bottle of wine in here anyway*.

Ali explained that being in control and being confident that she could say “stop” and know that her partner “will stop, no problem” was extremely important as she worries about “the sex part” of her relationship because:

*it does hurt a lot because of the place of the endometriosis… like when he inserts his thing, it hurts because it touches it, or when you have an orgasm… and that hurts. I think communication is so important. It doesn't worry me as such that he won't listen to me, because he does listen to me. I'm like, “We need to stop. It hurts.” He will stop, no problem (Ali)*.

### 3.3. It's OK to be vulnerable with each other: we can talk it through

The young adults alluded to the usual vulnerabilities and uncertainties that occur in romantic relationship. This was typified by Ellie who explained “romance comes [with] a lot of vulnerability and stuff”. However, pain and/or condition-related disabilities and limitations generated additional tensions, concerns, and vulnerabilities particularly for the young adults with chronic pain. Grace explained that living with pain meant exposing yourself to “repetitive vulnerability” as you had to provide explanations and entering a romantic relationship was fraught with a sense that “it might not work out” due to the pain.

Some young adults with chronic pain voiced a degree of dread for the first time their partner would see them in pain, as Ellie further explained, “absolutely at some point, I’ll have to let him see me vulnerable like that, which is quite scary but it has to happen eventually I think”. Vulnerability was heightened when pain was intense and/or threatening. Overwhelming pain sometimes meant that vulnerability could extend beyond the individual to the couple making them feel “[like] a cloud hanging over our heads” (Flo). Whilst this sense of enhanced vulnerability was distressing and fear-inducing for many young adults, for others, this sense of “learning to be vulnerable” (Flo) was liberating as it resulted in being comfortable “asking for help…[and] being okay to talk about how I'm feeling” (Flo).

All couples talked of how open their relationships were and how talking things through was an important and critical reciprocal component in making relationships work. While some couples were “always quite good with communication” (Christie) they also recognized that pain meant they had “got better……[and had added] another kind of layer” (Charlie^P^). Others stated that they had become “more confident to share things with each other, and share how I feel, because nothing else seems as big as an issue [as pain]” (Indi). This required work, and as Flo admitted, she had learned to think:

*‘okay, no’, like, that's not how you communicate, that's not alright, just take a step back, you're feeling this way because of the pain and it's not fair to take that out on someone else (Flo)*.

Being open and talking was important as it was the basis of reciprocal trust and of accepting and receiving help:

*It makes me feel good that she [Flo] trusts me to kind of ask me for help and rely on me for that stuff. And I kind of am the same way with her. I don't necessarily talk about certain things with my friends or my family, but I always talk about anything with her (Finlay^P^)*.

Gabriel^P^ talked of how “being honest and open [means] we've mastered the art of knowing how each other feel and working around that”. Talking was not the only aspect of communication that the young adults mastered, the partners learned to read their partners' non-verbal cues. Charlie^P^ explained that he could “see” Christie's pain:

*“… in your face and how you walk and get around. Even just going up the stairs, if you're having a bad day, you can tell, because you're going slower and that (Charlie^P^)*.

Disclosing pain was a specific and sometimes trickier aspect of being open with their partners. Disclosure was an ongoing process as different aspects of the impact of pain exerted an influence or created an imperative to share. For some young adults “first disclosure” was relatively simple as it just happened. Daisy did not recall specially talking about it, but her partner was made aware through “kind of being exposed to my routine of looking after my back after hockey… it wasn’t like ‘look you’re going to have to help me in this relationship’”. Indi and Brianna explained that circumstances had impelled them to disclose. Indi did not have the luxury of picking the right time as, early on in her relationship she needed surgery, and this forced the “what is actually wrong” conversation with her partner. Brianna's disclosure was impelled by the sense she “had to” when she about to engage in a social activity she knew she would struggle with:

*when I first met her [Billie], it wasn't a big thing I shared….I kind of had to. Because we went on a four-mile walk along the coast and I was in so much pain by the end of that, that, if I hadn't said anything beforehand, she would have been really worried (Brianna)*.

For other young adults, disclosing pain and/or their conditions involved weighing up the risks and vulnerabilities associated with disclosure:

*So, it's like, how do I go about telling somebody I've got all these illnesses and how do I go about saying that the pain I'm in or the other symptoms means that I might not be able to do certain things or go to certain places and …., you don't want to be a burden to that person (Ellie)*.

Ali who carefully thought through “should I disclose this, should I not?” was clear that a person's reaction to disclosure gave insight into their worth as a potential partner noting that:

*if they are like, “Oh, well then, I don’t want to be with you. You are going to be in pain all day”…. that means they are wee bastards (Ali)*.

Just as disclosure was rarely a “formal conversation” (Evan^P^) it also did not involve disclosing everything in one go:

*you'd sort of mentioned stuff and had stuff on your profile, [I'd] briefly Google it and get a rough idea of what the stuff was …. I probably…had a general feel [after] we'd been talking for eight months….but wouldn't have known everything in detail (Evan^P^)*.

As with Evan^P^, some partners wanted to know more and Harry^P^ explained he was “always curious about it, so I would be asking questions all the time” and Indi explained that she regularly “shares articles and other things to help keep him up to date and to inform him [her partner]”. There was a reciprocity in information exchange.

Perhaps the most heightened disclosures for the young adults with chronic pain were those ones associated with sex. However, as the following exchange shows things went better than Grace had expected, with humor playing a role in the disclosure.

*Grace: I'm quite upfront person. I don't really like you know, I'm not shy to say this or this. But you know, there's still that. Oh, God, I've got to, you know, how do you even start this conversation? …… it's a bit of a weird thing to talk about over cornflakes…*

*Gabriel^P^: Crunchy nut [cornflakes] (laughs) …*

*Grace: I was so surprised by like, you were just so on board from day one…*

*Gabriel^P^: Well, I did quite like you to be fair…*

*Grace: Yeah, but you didn't even like, blink at it, you weren't even like taken aback*.

Although disclosure can be feared, it was apparent that within a safe, secure, connected, and reciprocal relationship even the most difficult subjects could be addressed.

### 3.4. You can't see over the horizon: hopes and fears for the future

The young adults seemed hopeful for their futures. Their relationships were strong and they were managing to live good lives as couples, despite the interference of pain. Flo and Finlay^P^ were particularly hopeful as a recent surgery had been successful and reduced Flo's previous limitations. Flo acknowledged they were

*“in a new kind of [exciting] phase of life, where we're able to go out, and do things. I've been very into figuring out what my body can do now like yeah, in activities”*.

Christie had recently started getting support to live with her pain and she explained “it has helped us worry less for the future, because the idea of this is that eventually I will get better. I might not return to my old self, but I should be able to improve”. For others, hope for the future was generated through social comparisons with known individuals. For example, Dylan^P^ predicted his partner's pain trajectory would be like her father's as she is “basically a female genetic copy of him…[and] nothing's stopping him from doing anything”.

However, to a greater or lesser extent couples worried about pain and other symptoms and impacts getting worse. However, as Hazel suggested, worrying about the long-term was pointless as “you can’t predict what [chronic pain's] going to be like in 10 or 15 years, you can only take a guess of what it's going to be like in two days or a week”. Grace echoed the unpredictability and “uncertainty” about the future and potential progression of her condition, noting that that this uncertainty extended even to the “specialists [who] are the most knowledgeable … don't know”.

Although some young adults with chronic pain worried about how they might “handle” (Ali) worsening pain, typically, they talked about their worries about the impact on their partner. However, partners tended to be more pragmatic, as Evan^P^ explained: “I wouldn’t want anything that you have to deal with to get worse… [although] we [would] be able to deal with it as we go”. This pragmatic approach to life and living with pain was evident in terms of worries about worsening future pain:

*I think it's a worry, I think knowing that it's on the horizon. It's like, you know, like baldness running in my family. It's gonna happen. And I'm anxious for it, but we're gonna have to deal with when we get to it (Gabriel^P^)*.

Some young adults with chronic pain, like Ellie, expressed concerns about being a future burden on their partner.

*So, I think … as you get older, your body kind of breaks down…. it's not as good as it used to be. And, if I'm this age, 25, and my body does all this stuff now, it does make you wonder is it going to get so much worse and how is he going to cope with it (Ellie)*.

However, their partners seemed more sanguine about the impact on them focusing their concerns on the pain rather than the impact on their longer-term relationship, as Charlie^P^ explained “not really [worries]. I don’t like seeing (Christie in pain), but not in terms of the relationship” and providing reassurance that “I’m not just going to leave”.

Other worries such as employment were discussed. Brianna worried about full-time opportunities being limited by her pain-related fatigue, noting the balance between “making enough money to actually survive while also not wearing myself into the ground”. Hazel was concerned about the upcoming separation and reduction in support when she left to go to university; she was not looking forward to “being away from each other” and maybe only seeing each other every other weekend.

The other worries specifically related to the couples where pain was linked to a condition that affected or potentially affected fertility or meant sex was painful. For those young adults with concerns about fertility and having children, the “clock was ticking”. Ali looked to a future “as a team”, accepting “if we have to go through IVF we will go through IVF”. However, Ali was frustrated that her doctors would not do tests because she is “too young” proposing they would “wait until you are in your late 20s or your early 30s or maybe your 40s”. She saw these delays as problematic as she explained:

*I don't want to be older than 28 when I have children. I don't want to meet the 30 mark because I know what my energy levels are now, especially with chronic pain and that, what my energies will probably be. Because endometriosis, it progresses, right? (Ali)*.

Flo was also worried about the time window suggested by her gynecologist:

*So, she gave me a kind of a window to start trying 25, 25 to 27, which is sudden, I mean, that's only three years away for us. I think it's kind of two folded, there's the worry about not being able to have biological children, and then there's the worry about that so soon, you can hope we're young, [but] we don't have any money (Flo)*.

Finlay^P^ acknowledged that Flo “worries about if we can have kids in the future” but also noted that “for all I know, I'm infertile, and we can't have kids [because of me]”. Although Indi had worried that she and her partner “would have to end [the relationship]” if she could not have children, she had recently found out that she was pregnant and explained that they felt “so lucky”.

Grace openly expressed a worry for the future of their relationship and whether she would, due to her pain, be “[able to satisfy] that side of life…. fulfil something in their life, you know, the sex area that they felt needs to get filled”. She went on to reflect that “I think it'd be totally fair enough to go look for that elsewhere. But obviously, that is a sad thing. And, you know, something that isn't my fault, either”.

## 4. Discussion

In this qualitative exploration of romantic relationships among those with chronic pain and their partners, reciprocity was recognized and understood by couples as a key part of their relationship, and it ran throughout their narratives. For example, couples talked about managing the chronic pain “together”, highlighted shared interactions and shared exchanges, where both parties respectfully behaved and responded to each other, and discussed vulnerability. Such discourse shows a mature approach to emotional involvement by the individuals in the relationship: they had as much concern for themselves as they did for each other, and they were able to maintain a distinct sense of self within the partnership. This emotional maturity is of interest in relation to their stage of development (young adulthood), which is seen as a developmental stage about attaining, among other factors, self-efficacy, integrity, trustworthiness, caring for others, connectedness, and sharing intimacy (48). The young adults appeared to be achieving these developmental tasks. Further, the young adults in our study understood the relationship to be a partnership rather than an alliance: there was no asymmetry, where one member was seen to be the care giver and the other member the care recipient. While asymmetry in the caregiver-care recipient relationship is found among older age partners when one partner experiences chronic illness (49) and caregiver burden is shown to correlate with pain intensity in adults (50), we did not find this among the young adults in the current study.

Reciprocity—a process of giving and taking (22)—includes an exchange of emotions, thoughts, and behavior and is a central factor in romantic relationships among adults from diverse backgrounds (25–28). Missing from the literature about reciprocity, until now, has been an exploration of how young adults with long-term health conditions and their partners overcome challenges in sustaining romantic relationships. Findings from our study suggest that reciprocity is the backbone of those romantic relationships. Up until now, it has not been clear on what terms romantic relationships work between young adults where one partner experiences chronic pain. It seems from our study that reciprocity, and the belief that chronic pain does not define the relationship, are crucial to relationship success among young adults. Couples highlighted that, even during challenges associated with caregiving, their commitment to each other, their shared values and a sense of hope and optimism about the future remained intact and moved them forward. This commitment is seen in strong relationships in older adults who are able to navigate the demands associated with living with a long-term condition (31). The helping motivation of partners of older adult chronic pain patients has been shown to positively impact on basic psychological needs of the person in pain (51). Our findings suggest that is also evident in the romantic relationships of young adults.

The sense of hope for the future that pervaded the conversations with and between the couples perhaps reflects the developmental stage of young adulthood—where they were looking to the future and excited by the sense of the new possibilities that their relationships were creating. There is literature, including systematic reviews and meta-analyses, that address hope and/or optimism and show that these can be protective or buffering factors in relation to chronic pain (52–54) and can support adjustment (55) or adaptation (54) to chronic pain. However, none of this hope-related work has focused on young adults.

### 4.1. Strengths, limitations, and future work

Our sample provided us with couples who had been together for some time [only one couple had been together for less than 12 months and most (*n* = 5) had been together for more than 3 years]. Thus, the young adults in our study were not in relatively new relationships, so the views of young adults in more established romantic relationships are presented here. In future work, researchers will want to explore more extensively how time together impacts the narratives of romantic couples where one experiences chronic pain. Additionally, chronic pain was integrated during the forming of these romantic relationships which may be different than when chronic pain disrupts already formed romantic relationships where patterns of being a couple are already established.

Although there was sexual diversity across the sample, and the couples were in relatively established relationships, only one couple was recruited from Canada, perhaps limiting transferability to the Canadian context, although culturally the countries are not dissimilar. Most young adults in pain (*n* = 6) were ages 22 years or older, so there is less representation from younger participants. All but one young person with chronic pain had experienced pain for 5 years or more, so we are missing the experience of young adults in the early years of chronic pain experience. Additionally, all our participants presented themselves as being in a happy and satisfying relationship. This limits the transferability of findings to couples in less fulfilling relationships and this should be an area for future research.

The detailed demographic information about the young adults in chronic pain was generated within the survey conducted during the previous phase of the study. However, similarly detailed information (e.g., age or gender) of the partners was not collected. This limits the findings. However, in relation to age, the partners had to be 18 years or older.

Further, while it is likely that individual differences make important contributions to successful romantic relationships for young adults with chronic pain (18), we were not able to examine those in depth. We included couples from a range of diverse sexualities, but we do not know whether other individual differences have an impact on how partners describe their relationship when one member reports chronic pain. Other important individual differences to explore in future work include mental health challenges and/or financial insecurity, which we know impact time devoted to the relationship (56, 57) and additional pressures that affect reports of relationship quality (58).

It is noteworthy that all the participants with chronic pain were biologically female. Thus, the findings presented here represent those from biological females with chronic pain, and, thus, we do not claim to provide information on how men with chronic pain and their partners experience and define their relationships. Given that, future work will want to explore how young men with chronic pain and their partners navigate romantic relationships.

Future work needs to explore reciprocity, hope, and optimism within romantic relationships with a specific focus on the young adult population. Additionally, future work in the context of chronic pain in young adult should helpfully focus on examining a range of individual, interpersonal, and cultural factors that may influence romantic relationships in young adulthood (59). Examples to consider include family and peer relationships earlier in life (3), as initial work shows that having relational support is particularly important for emerging adults in LGBT+ relationships (60).

We acknowledge the limitations of our data, but we offer information directly from young adults and their partners about important characteristics of their romantic relationships, with reciprocity being a common feature for couples of different sexual orientations. The voice of partners of those with chronic pain is documented, offering an important perspective that could inform discussions between health professionals and young adults in pain.

### 4.2. Implications

Despite its importance, the impact of chronic pain on the romantic relationships of young adults and how they sustain those relations is poorly understood. Previous quantitative findings showed that young adults with chronic pain were more likely than their peers without chronic pain to report poorer quality romantic relationship (18). What we have shown in the current study is that this is not the case for *all* young adults, and the narratives provided here highlight important avenues for intervention. For example, the focus on reciprocity within relationships is likely a significant discussion topic for those transitioning to adult care from paediatric/child health care and that disclosure is welcomed by partners. It may also be reassuring to adolescents with pain that pain is not inevitably a limiting factor in romantic relationships: some reports (61) show that young adults wait until their pain is resolved before engaging in a romantic relationship, but it seems that is not necessarily needed.

Our findings show that young adults can experience happy and satisfying romantic relationships and they reveal some of the ways in which these relationships were built, for example through partnership, a sense of reciprocity, loving in everyday acts, and concerned supportiveness. These findings could be the basis for intervention work either preparing adolescents and young adults about what to expect and how to achieve a happy romantic relationship, or for couples who need support because their relationship is uncertain, unhappy, or not satisfying. Such ideas fit well with recent work on hope (53) and work that proposes a shift to involving partners as co-participants and beneficiaries in pain interventions, aiming to improve well-being and reduce relationship distress in couples where one or both experience chronic pain (62).

## 5. Conclusions

Hopefulness and reciprocity were key to the stories shared by the young adults in this study. Despite the challenges and limitations imposed by chronic pain, their relationships were characterized by partnership and reciprocity, and they were able to be vulnerable with each other and offer each other support.

## Data Availability

The raw data supporting the conclusions of this article will be made available by the authors, without undue reservation.

## References

[1] van Harmelen AL Kievit RA Ioannidis K Neufeld S Jones PB Bullmore E Adolescent friendships predict later resilient functioning across psychosocial domains in a healthy community cohort. Psychol Med. (2017) 47(13):2312–22. 10.1017/S0033291717000836 28397612 PMC5820532

[2] Kiecolt-Glaser JK Newton TL. Marriage and health: his and hers. Psychol Bull. (2001) 127(4):472–503. 10.1037/0033-2909.127.4.472 11439708

[3] Rauer AJ Pettit GS Lansford JE Bates JE Dodge KA. Romantic relationship patterns in young adulthood and their developmental antecedents. Dev Psychol. (2013) 49(11):2159–71. 10.1037/a0031845 23421803 PMC3830676

[4] The Society for Adolescent Health and Medicine. Young adult health and well-being: a position statement of the society for adolescent health and medicine. J Adolesc Health. (2017) 60(6):758–9. 10.1016/j.jadohealth.2017.03.021 28532650

[5] Figueroa JM DeLuca Bishop HK Baker EA. Using a socio-ecological framework to understand romantic relationship satisfaction among emerging adults during the COVID-19 pandemic. Emerg Adulthood. (2022) 10(6):1561–73. 10.1177/21676968221124266 PMC9434192 38603198

[6] Boisvert S Poulin F. Navigating in and out of romantic relationships from adolescence to emerging adulthood:distinct patterns and their correlates at age 25. Emerg Adulthood. (2017) 5(3):216–23. 10.1177/2167696816675092

[7] Dougall M Konantambigi RM Khanna R. Nature of romantic relationship in committed emerging adults: exploring challenges and resilience. Psychol Stud (Mysore). (2022) 67(4):537–48. 10.1007/s12646-022-00692-5

[8] Uçar S. Initiating, maintaining and terminating a romantic relationship during emerging adulthood in Turkey. Curr Psychol. (2022) 41(2):1015–25. 10.1007/s12144-021-01431-1

[9] Shulman S Connolly J. The challenge of romantic relationships in emerging adulthood:reconceptualization of the field. Emerg Adulthood. (2013) 1(1):27–39. 10.1177/2167696812467330

[10] Jordan A Carter B Forgeron P Fournier K Sanders K. Romantic relationships in young people with long-term health conditions: a scoping review. J Pediatr Psychol. (2020) 46:264–79. 10.1093/jpepsy/jsaa106 33306805

[11] Treede RD Rief W Barke A Aziz Q Bennett MI Benoliel R Chronic pain as a symptom or a disease: the IASP classification of chronic pain for the international classification of diseases (ICD-11). Pain. (2019) 160(1):19–27. 10.1097/j.pain.0000000000001384 30586067

[12] King S Chambers CT Huguet A MacNevin RC McGrath PJ Parker L The epidemiology of chronic pain in children and adolescents revisited: a systematic review. Pain. (2011) 152(12):2729–38. 10.1016/j.pain.2011.07.016 22078064

[13] Kashikar-Zuck S Parkins IS Ting TV Verkamp E Lynch-Jordan A Passo M Controlled follow-up study of physical and psychosocial functioning of adolescents with juvenile primary fibromyalgia syndrome. Rheumatology (Oxford). (2010) 49(11):2204–9. 10.1093/rheumatology/keq254 20688804 PMC2954368

[14] Murray CB de la Vega R Murphy LK Kashikar-Zuck S Palermo TM. The prevalence of chronic pain in young adults: a systematic review and meta-analysis. Pain. (2022) 163(9):e972–e84. 10.1097/j.pain.0000000000002541 34817439

[15] Brown D Schenk S Genent D Zernikow B Wager J. A scoping review of chronic pain in emerging adults. Pain Rep. (2021) 6(1):e920. 10.1097/PR9.0000000000000920 34712883 PMC8546842

[16] Mills SEE Nicolson KP Smith BH. Chronic pain: a review of its epidemiology and associated factors in population-based studies. Br J Anaesth. (2019) 123(2):e273–e83. 10.1016/j.bja.2019.03.023 31079836 PMC6676152

[17] Yong RJ Mullins PM Bhattacharyya N. Prevalence of chronic pain among adults in the United States. Pain. (2022) 163(2):e328–e32. 10.1097/j.pain.0000000000002291 33990113

[18] Murray CB Groenewald CB de la Vega R Palermo TM. Long-term impact of adolescent chronic pain on young adult educational, vocational, and social outcomes. Pain. (2020) 161(2):439–45. 10.1097/j.pain.0000000000001732 31651579 PMC7001863

[19] Pooleri A Yeduri R Horne G Frech A Tumin D. Pain interference in young adulthood and work participation. Pain. (2023) 164(4):831–7. 10.1097/j.pain.0000000000002769 36048525

[20] Cartwright T Fraser E Edmunds S Wilkinson N Jacobs K. Journeys of adjustment: the experiences of adolescents living with juvenile idiopathic arthritis. Child Care Health Dev. (2015) 41(5):734–43. 10.1111/cch.12206 25287720

[21] Tankha H Caño A Dillaway H. Now I have hope": rebuilding relationships affected by chronic pain. Fam Syst Health. (2020) 38(1):51–6. 10.1037/fsh0000472 32202833 PMC7101054

[22] Buunk BP Schaufeli WB. Reciprocity in interpersonal relationships: an evolutionary perspective on its importance for health and well-being. Eur Rev Soc Psychol. (1999) 10(1):259–91. 10.1080/14792779943000080

[23] Carlson W Rose AJ. The role of reciprocity in romantic relationships in middle childhood and early adolescence. Merrill Palmer Q. (2007) 53(2):262–90. 10.1353/mpq.2007.0008

[24] Couture S Fernet M Côté P-B. Interaction patterns in adolescent romantic relationships: a typological analysis. J Res Adolesc. (2020) 30(3):706–20. 10.1111/jora.12553 32129555

[25] Lavner JA. Relationship satisfaction in lesbian couples: review, methodological critique, and research agenda. J Lesbian Stud. (2017) 21(1):7–29. 10.1080/10894160.2016.1142348 27557338

[26] Sabour Esmaeili N Schoebi D. Research on correlates of marital quality and stability in muslim countries: a review. J Fam Theory Rev. (2017) 9(1):69–92. 10.1111/jftr.12181

[27] Shpigelman C-N Vorobioff M. Romantic relationship and psychological wellbeing: the experiences of young individuals with visual impairment. Disabil Rehabil. (2021) 43(9):1228–36. 10.1080/09638288.2019.1661036 31491358

[28] Walker RB Luszcz MA. The health and relationship dynamics of late-life couples: a systematic review of the literature. Ageing Soc. (2009) 29(3):455–80. 10.1017/S0144686X08007903

[29] Mitchell MM Isenberg SR Maragh-Bass AC Knowlton AR. Chronic pain predicting reciprocity of support among vulnerable, predominantly African-American persons living with HIV/AIDS. AIDS Behav. (2018) 22(6):2002–7. 10.1007/s10461-017-1775-9 28451889 PMC5659968

[30] Lyons KS Gorman JR Larkin BS Duncan G Hayes-Lattin B. Active engagement, protective buffering, and depressive symptoms in young-midlife couples surviving cancer: the roles of age and sex. Front Psychol. (2022) 13:816626. 10.3389/fpsyg.2022.816626 35250747 PMC8891218

[31] Weitkamp K Feger F Landolt SA Roth M Bodenmann G. Dyadic coping in couples facing chronic physical illness: a systematic review. Front Psychol. (2021) 12:722740. 10.3389/fpsyg.2021.722740 34759866 PMC8573212

[32] Ickes W. Subjective and intersubjective paradigms for the study of social cognition. New Rev Soc Psychol. (2002) 1(112–121).

[33] Simpson JA Oriña MM Ickes W. When accuracy hurts, and when it helps: a test of the empathic accuracy model in marital interactions. J Pers Soc Psychol. (2003) 85(5):881–93. 10.1037/0022-3514.85.5.881 14599251

[34] Harker Tillman K Brewster KL Valle Holway G. Sexual and romantic relationships in young adulthood. Annu Rev Sociol. (2019) 45(1):133–53. 10.1146/annurev-soc-073018-022625

[35] Campbell MS Gray A Wiebe DJ Berg CA. Understanding the roles of romantic partners and parents in the management of type 1 diabetes in emerging adults. Diabetes Spectr. (2022) 35(1):66–75. 10.2337/ds21-0011 35308157 PMC8914585

[36] de Grey RG K Berg CA Campbell MS Munion AK Luyckx K Raymaekers K Diabetes support from romantic partners during early emerging adulthood. J Behav Med. (2022) 45(4):558–70. 10.1007/s10865-021-00271-5 35066695

[37] Gomez-Lopez M Viejo C Ortega-Ruiz R. Well-being and romantic relationships: a systematic review in adolescence and emerging adulthood. Int J Environ Res Public Health. (2019) 16(13):2415. 10.3390/ijerph16132415 31284670 PMC6650954

[38] Mackenzie N Knipe S. Research dilemmas: paradigms, methods and methodology. Issues Educ Res. (2006) 16(2):193–205.

[39] Tong A Sainsbury P Craig J. Consolidated criteria for reporting qualitative research (COREQ): a 32-item checklist for interviews and focus groups. Int J Qual Health Care. (2007) 19(6):349–57. 10.1093/intqhc/mzm042 17872937

[40] Malterud K Siersma VD Guassora AD. Sample size in qualitative interview studies: guided by information power. Qual Health Res. (2016) 26(13):1753–60. 10.1177/1049732315617444 26613970

[41] Sipes JBA Roberts LD Mullan B. Voice-only skype for use in researching sensitive topics: a research note. Qual Res Psychol. (2019) 19:1–17. 10.1080/14780887.2019.1577518

[42] Harper D. Talking about pictures: a case for photo elicitation. Vis Stud. (2002) 17(1):13–26. 10.1080/14725860220137345

[43] McConnell-Henry T Chapman Y Francis K. Member checking and heideggerian phenomenology: a redundant component. Nurse Res. (2011) 18(2):28–37. 10.7748/nr2011.01.18.2.28.c8282 21319482

[44] Braun V Clarke V. Reflecting on reflexive thematic analysis. Qual Res Sport Exerc Health. (2019) 11(4):589–97. 10.1080/2159676X.2019.1628806

[45] Conroy SA. A pathway for interpretive phenomenology. Int J Qual Methods. (2003) 2(3):36–62. 10.1177/160940690300200304

[46] Smith J Osborn M. Interpretative phenomenological analysis. In: Smith J, editors. Qualitative psychology: A practical guide to research methods. London: Sage (2003). p. 51–80.

[47] Kitto SC Chesters J Grbich C. Quality in qualitative research. Med J Aust. (2008) 188(4):243–6. 10.5694/j.1326-5377.2008.tb01595.x 18279135

[48] Scales PC Benson PL Oesterle S Hill KG Hawkins JD Pashak TJ. The dimensions of successful young adult development: a conceptual and measurement framework. Appl Dev Sci. (2016) 20(3):150–74. 10.1080/10888691.2015.1082429 30344455 PMC6176765

[49] Faronbi JO Faronbi GO Ayamolowo SJ Olaogun AA. Caring for the seniors with chronic illness: the lived experience of caregivers of older adults. Arch Gerontol Geriatr. (2019) 82:8–14. 10.1016/j.archger.2019.01.013 30710847 PMC6459393

[50] Tsuji H Tetsunaga T Tetsunaga T Misawa H Oda Y Takao S Factors influencing caregiver burden in chronic pain patients: a retrospective study. Medicine (Baltimore). (2022) 101(39):e30802. 10.1097/MD.0000000000030802 36181114 PMC9524903

[51] Kindt S Vansteenkiste M Brenning K Goubert L. The effects of Partners’ helping motivation on chronic pain Patients’ functioning over time. J Pain. (2019) 20(3):348–57. 10.1016/j.jpain.2018.09.008 30291905

[52] Boselie JJLM Peters ML. Shifting the perspective: how positive thinking can help diminish the negative effects of pain. Scand J Pain. (2023). 10.1515/sjpain-2022-0129 36803855

[53] Katsimigos A-M O’Beirne S Harmon D. Hope and chronic pain—a systematic review. Ir J Med Sci. (2021) 190(1):307–12. 10.1007/s11845-020-02251-1 32451764

[54] Shanahan ML Fischer IC Hirsh AT Stewart JC Rand KL. Hope, optimism, and clinical pain: a meta-analysis. Ann Behav Med. (2021) 55(9):815–32. 10.1093/abm/kaab001 33580660

[55] Or DYL Lam CS Chen PP Wong HSS Lam CWF Fok YY Hope in the context of chronic musculoskeletal pain: relationships of hope to pain and psychological distress. Pain Rep. (2021) 6(4):e965. 10.1097/PR9.0000000000000965 34712887 PMC8547930

[56] Braithwaite S Holt-Lunstad J. Romantic relationships and mental health. Curr Opin Psychol. (2017) 13:120–5. 10.1016/j.copsyc.2016.04.001 28813281

[57] Braithwaite SR Delevi R Fincham FD. Romantic relationships and the physical and mental health of college students. Pers Relatsh. (2010) 17(1):1–12. 10.1111/j.1475-6811.2010.01248.x

[58] LeBaron AB Curran MA Li X Dew JP Sharp TK Barnett MA. Financial stressors as catalysts for relational growth: bonadaptation among lower-income, unmarried couples. J Fam Econ Issues. (2020) 41(3):424–41. 10.1007/s10834-020-09666-z

[59] Uçar S Demir İ. Exploring romantic relationship patterns among turkish emerging adults: a grounded theory study. Emerg Adulthood. (2023) 11(1):88–102. 10.1177/21676968221094751

[60] Feiring C McMahon E Gall Z. Emerging adult LGB+ romantic relationships: narratives about met and unmet relationship needs. J Homosex. (2022):1–27. 10.1080/00918369.2022.2106807 35930805

[61] Forgeron PA McGrath P Stevens B Evans J Dick B Finley AG Social information processing in adolescents with chronic pain: my friends don't really understand me. Pain. (2011) 152(12):2773–80. 10.1016/j.pain.2011.09.001 21963240

[62] Tankha H Caño A Corley A Dillaway H Lumley MA Clark S. A novel couple-based intervention for chronic pain and relationship distress: a pilot study. Couple Family Psychol. (2020) 9(1):13–32. 10.1037/cfp0000131 34017649 PMC8132556

