# Supplementary material for: A shared love: reciprocity and hopefulness in romantic relationships of young adults with chronic pain
Source: Front Pain Res (Lausanne). 2023 Jun 14;4:1179516. doi: 10.3389/fpain.2023.1179516 (PMC10303136; doi:10.3389/fpain.2023.1179516)
Supplement: Supplementary file 1 [file Datasheet1.pdf]

The Chronic Pain and Romantic Relationships Study Interview Guide

1. Opening section

The interviewer summarises the study, giving the participants an opportunity to ask questions, then gaining consent (as per procedure). We will reassure the participant that there are no right or wrong answers and that we are just interested in their perceptions and experiences.

We will reiterate the details about confidentiality, anonymity, withdrawal from study and withdrawal of data as part of our consent procedure.

2. Main section

Participants can choose either the photo-elicitation option or a more traditional semi-structured interview.

Photo-elicitation option

The interviewer will invite the participants (YPwCP and their partner) to talk about the 3-4 images that they have each selected to share. These images can be shared during the interview or in advance via secure file sharing.

Using open questioning and the photographs/images as prompts, the researcher will engage the participants in conversation to elicit their perceptions and experiences associated with navigating their romantic relationship. Although the interviewer will mostly be relying on the photographs and images to trigger conversation, they will also have topic areas that they can address. In specific, we hope to cover the:

- Overview of the relationship (how they met, how long they have been together)
- When you met did [name of person] have chronic pain?
    - If [name of person] did not have chronic pain and it developed/was diagnosed once you had met, what was that like? (e.g., challenges/easy/hard/impact on relationship; Did it make a difference to relationship?)
- If [name of person] already chronic pain how was that 'disclosed' to partner?
    - How easy/hard was this? Did it make a difference to relationship?
- If [name of person] already had chronic pain, when and how was chronic pain disclosed to partner (challenges/easy/hard, impact on relationship)?
- How they describe their romantic relationship (e.g., sense of connection, feeling loved).
- Does the experience of living with/living with a partner who has chronic pain bring you any benefits as a couple or as individuals (such as a sense of closeness)
- Negative things and challenges that chronic pain may bring to them as individuals/their relationship (e.g., worried about rejection).

- Worries or concerns (things that can be stressful) related to chronic pain and their relationship (e.g., worries about future, impact on how they feel about themselves, their well-being).
- Solutions or strategies they have adopted to manage the challenges (e.g., pacing, changes to activities).

Traditional semi-structured interview

The interviewer will invite the participants (YPwCP and their partner) to talk about their perceptions and experiences associated with navigating their romantic relationship. The interviewer will be covering the same topic areas indicated above through using the following questions and prompts (although they will not necessarily be addressed in this order and the phrasing may change in response to individual contexts.

- Please tell me about your relationship
  - How did you meet? How long they have you been together?
- When you met did [name of person] have chronic pain?
  - If [name of person] did not have chronic pain and it developed/was diagnosed once you had met, what was that like?(e.g., challenges/easy/hard/impact on relationship; Did it make a difference to relationship?)
- If [name of person] already chronic pain how was that 'disclosed' to partner?
  - How easy/hard was this? Did it make a difference to relationship?
- How would you describe your romantic relationship?
  - e.g., sense of connection, feeling loved.
- What sorts of benefits for your relationship, if any, have resulted from living with chronic pain?
  - e.g., sense of closeness, not worrying about 'small' stuff
- What sorts of challenges and negative impacts, if any, have resulted from one of you having chronic pain?
  - e.g., worried about rejection, that pain could get worse.
- What worries or concerns (things that can be stressful) do you have as individuals/partners related to chronic pain and your relationship?
  - e.g., worries about future, impact on how they feel about themselves, their well-being.
- What solutions or strategies have you adopted as individuals/partners to manage these challenges?
  - e.g., pacing, changes to activities.

3. Closing section

This will involve thanking the participants, checking that they are OK and asking if they have any other questions. The interviewer will remind them that they will send them a **Helpful Information Sheet** which will confirm that we are grateful for their participation and which will direct them to well-being and pain-related resources that provide help, advice and support, as needed.
